# Fifteen years in, what next for *PLOS Biology*?

**DOI:** 10.1371/journal.pbio.3000049

**Published:** 2018-10-15

**Authors:** The PLOS Biology Staff Editors

**Affiliations:** Public Library of Science, San Francisco, California, United States of America, and Cambridge, United Kingdom

## Abstract

As we celebrate our anniversary, the PLOS Biology editors discuss recent initiatives taken by the journal (meta-research, complementary research policy, preprint posting, short reports, methods and resources, data policy, protocols.io) and look ahead to the next fifteen years.

*PLOS Biology* marked its fifteenth anniversary on October 13 (Fig 1). The year we published our first issue, 2003, Europe launched its first voyage to Mars, the SARS epidemic spread through 26 countries [1], and the Human Genome Project published all the nucleotide base pairs in our DNA. Our launch predated Facebook, YouTube, Twitter, smart phones and tablets. In the US at the time, 43% of households had dial-up, ‘slow internet’, 38% had no internet and only 19% had home broadband; only 1% of music sales were digital; and 37% of households had no cell/mobile phone [2]. Yet the promise of digital technology to transform scientific communication was already apparent. “Communication among scientists has undergone a revolution in the last decade, with the movement of scientific publication to a digital medium and the emergence of the Internet as the primary means for distributing information,” the PLOS founders wrote in our first issue [3]. “Millions of articles are, in principle, just a mouse-click away from our computers.”

**Fig 1:**
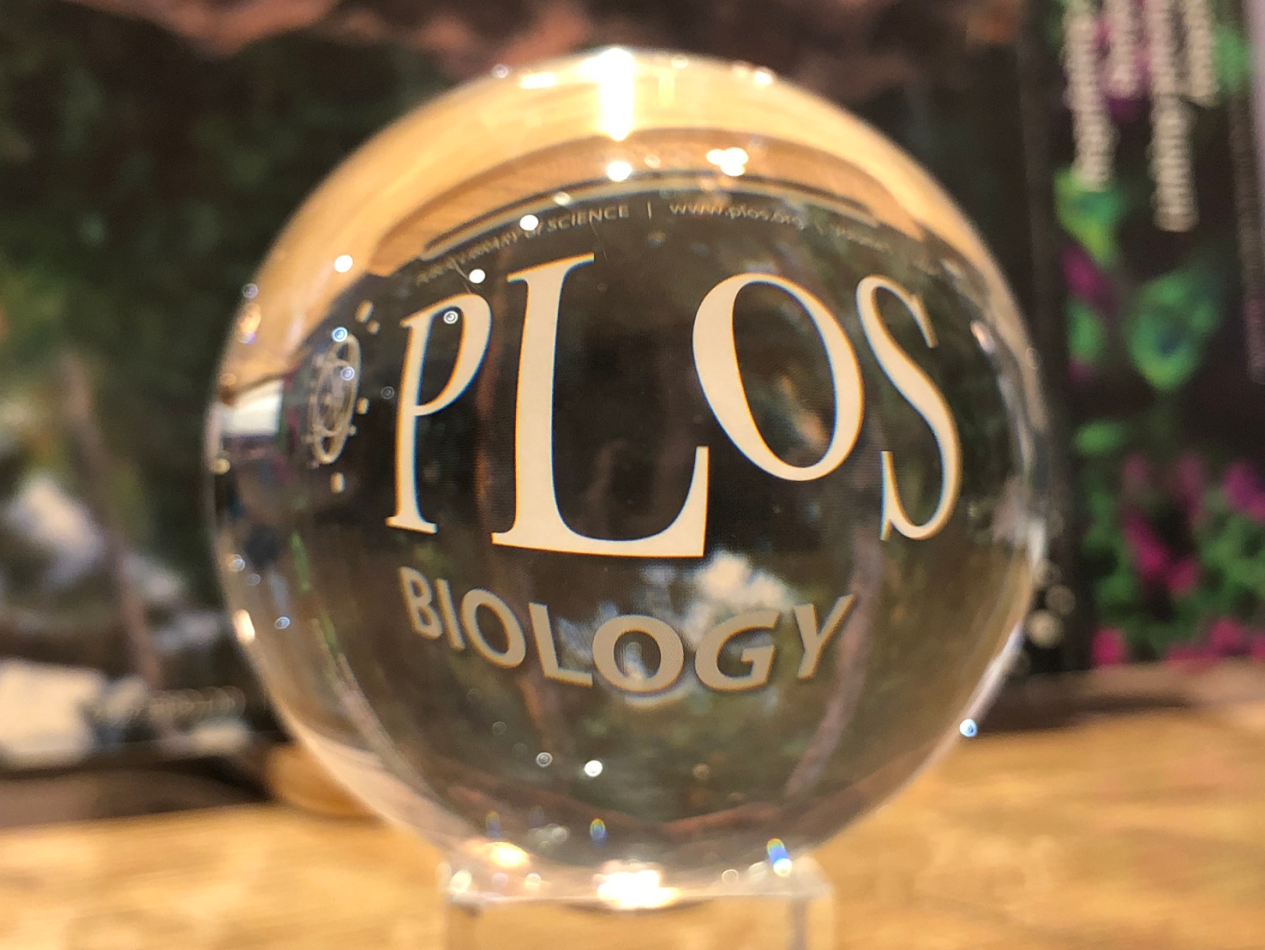
*PLOS Biology* celebrates fifteen exciting years. As we celebrate our anniversary, we recap here recent initiatives taken by the journal (Meta-Research, Short Reports, Methods and Resources, complementary research policy, preprint posting, data policy, protocols.io) and although we cannot predict the future, we look ahead to the next fifteen years.

## Early hopes

The hope was that the obvious benefits that digital technology offered by removing barriers to accessing scientific information would catalyse radical change in the scholarly publishing landscape. Progress in research publishing and dissemination, however, has not kept pace with advances in technology. As open access advocate John Wilbanks notes in the movie Paywall: The Business of Scholarship [4] (a very interesting watch if you have an hour to spare), “We can make cars that can drive; are you telling me that we can’t process the literature better?” *PLOS Biology* has achieved a lot in fifteen years, but it’s clear there is still much to be done (Box 1).

**Box 1. PLOS’s CEO, Alison Mudditt on *PLOS Biology*.**

In its first fifteen years, *PLOS Biology* has overturned the myth that an open access business model and top-quality science are mutually exclusive. It has also been instrumental in catalysing a range of important transformations in scientific communication from its rigorous implementation of PLOS’s data policy to expand the definition of what can and should be shared, to the new complementary research policy that showcases the value of replication. I’m proud that *PLOS Biology* has achieved all of this while also providing excellent service to its community of authors. As we look to the future, there is plenty more work to be done and *PLOS Biology* continues to lead the charge with the next wave of change: expanding our definitions of what should be published and how it should be assessed and curated.

Fifteen years ago, open access publishing was in its infancy. PLOS along with BioMed Central were making waves in the publishing industry, hoping that scientists and civic-minded publishers would embrace open access publishing as a public good. But the waves proved too small to create a groundswell. Subscription-based publishers reluctant to lose a steady source of income by converting to open access subverted the goals by simply adding open access options while enforcing paywalls to most scientific content. And decisions about the quality of research continue to rely on journal title as a proxy for value and impact. As a result, although more researchers are aware of open access, they continue to aspire to publish in journals that check career-advancement boxes, with little thought to how publisher business model affects the ability of science to progress. Initiatives like DORA—founded to identify ways to improve how research is assessed—have helped raise awareness of the fallibility and inappropriateness of metrics like the impact factor [5], yet the ingrained desire to publish in the ‘glamour mags’ persists. The revolution, it seems, has yet to happen.

Overturning entrenched norms, especially those backed by powerful interests, is never easy. But some developments offer reason for hope; Plan S in the European Union is an open access policy announced recently by a group of European national funders, with support from the European Commission and the European Research Council, which commits to eliminate paywalls in science; these funders will mandate that after 1 January 2020, access to research publications generated from all research that they fund must be fully and immediately open access [6]. Questions persist about article publication charges, who should cover the costs of publication, and whether there should be a cap on fees. Yet awareness of the value of open access is growing among these EU funders who will (rightly) claim a vested interest in the output of research that they’re funding. Such initiatives are a meaningful step in the right direction, making the long-awaited revolution more likely; we hope that globally other funders or coalitions will follow this encouraging lead.

## Evaluating the research process

Aside from influencing access to scientific research, publishers have the potential to change the scientific process itself. Much has been made in recent years about rigour and reproducibility in research; some have called this a “reproducibility, or replication crisis” [7], a characterization that some corporate and anti-science forces have weaponized and exploited to downplay evidence that threatens their interests. Science is naturally self-correcting over time with many results garnering support and being built upon by subsequent studies. That said, completing a precise replication is much more complex than may be apparent due to vagaries of experimental details and techniques. Another view is that replication challenges are an almost inevitable outcome given how scientists and their research are assessed; the warped incentive system often places a premium on publishing first, with minimal regard to the soundness of the data and study design. As a result, researchers can be rewarded with tenure and promotions not for the strength of their research but instead according to meaningless proxies for quality, such as the impact factor or title of the journals they publish in.

We knew there had to be a better way. In early 2016, *PLOS Biology* expanded its scope to publish ‘research into research’ in the form of Meta-Research Articles [8]. These articles are data-driven and include experimental and meta-analytical research that addresses issues related to the design, methods, reporting, and evaluation of research. We feel strongly that the best way to improve an imperfect situation is first to better understand the nature of the issue, and for this to happen ‘research into research’ needs a venue for publication and acceptance as a core branch of the scientific process.

## Establishing new norms

We’ve also attempted to address the counter-productive pressure for scientists to publish first by dispelling the negative connotations of being ‘scooped’; earlier this year *PLOS Biology* formalised a ‘complementary research’ policy that gives authors of a complementary study six months from the publication of the first article to submit their manuscript to *PLOS Biology* [9]. With this, the intense pressure of the race to publish is alleviated, and researchers—especially early career researchers (whose careers can be severely impacted by one such ‘scooping’ incident)—still have the opportunity to publish. This policy recognizes the value of independent replication of research findings rather than penalizing authors for lack of perceived novelty.

Alongside this, *PLOS Biology*, along with most of the other PLOS journals, now offers submitting authors the option of having us facilitate posting a preprint of their manuscript on bioRxiv. This journal-to-preprint model helps achieve much earlier open access to those research results while the manuscript is under review.

In the last year, we’ve also taken steps to acknowledge the inherent value of research outputs that are often overlooked by adding two new article types: ‘Short Reports’ and ‘Methods and Resources.’ The former validates publication of exploratory research without all the mechanistic bells and whistles, and the latter recognizes researchers who focus more on creating tools or datasets for their research community. With a view to better recognising other types of research outputs, *PLOS Biology* has also embraced and enforced the PLOS data policy [10] and we strongly encourage deposition of experimental protocols in protocols.io [11,12].

*PLOS Biology* has long distinguished itself by using a unique peer-review model that partners in-house professional editors with trusted advisors in the scientific community on every reviewed manuscript. We remain indebted to our partners on the Editorial Board who ensure an unbiased and expertly guided assessment and review process. Rather than looking for reasons to reject, our goal is to help researchers publish their quality science. When faced with opposing recommendations if appropriate we mediate cross-reviewer commenting, and we sometimes publish even if there are still open questions as long as any caveats are clearly stated in the manuscript or in an Editor’s Note [13–16]. As we have from the beginning, we aspire to work with and for scientists to smooth the path to publication.

## Crystal gazing

As we proceed through our teenage years, *PLOS Biology* is focussing on how we can address other research pain points, and how we fit into the current STM publishing ecosystem while simultaneously working towards open research. We’ve spent the last five years closely focusing on our editorial processes and improving author service. We’re committed to adapting to the needs of the community, innovating and experimenting in the publishing space, and redefining selectivity for publication assessment. In August this year, PLOS signed an open letter committing to publishing peer review reports for all PLOS journals [17]. What the future will bring for open access publishing remains uncertain, but *PLOS Biology* is poised, ready to adapt and evolve, and remains dedicated to the goal of accelerating progress in science and medicine by leading a transformation in research communication.

Meanwhile, to celebrate 15 years of *PLOS Biology*, we continue to publish high-quality, rigorous science, and we’ve been showcasing blog posts each month throughout 2018, featuring an article selected by one of our hard-working Editorial Board Members. Each post highlights that editor’s favourite article along with their commentary on its importance [18]. Take a look at the Collection [19].

In the first editorial of the inaugural issue, the *PLOS Biology* editors urged readers to help improve the journal to maximize the benefits derived from the time, money, effort and intellectual capital invested in the scientific process [20]. The editorial staff has changed but the sentiments have not. And we repeat that call now, when the very process and output of science is under attack in the United States and elsewhere. Open access, open science, and open research have the potential to create a powerful scientific and public resource. Working together, we can realize that goal.
